# Mucosal-Associated Invariant T Cells in Inflammatory Bowel Diseases: Bystanders, Defenders, or Offenders?

**DOI:** 10.3389/fimmu.2015.00027

**Published:** 2015-02-02

**Authors:** Emmanuel Treiner

**Affiliations:** ^1^Centre de Physiopathologie de Toulouse-Purpan (CPTP) INSERM UMR1043, Toulouse, France

**Keywords:** inflammatory bowel diseases, inflammation, innate lymphocytes, MAIT cells, cytokines

The quest for new therapeutics and better follow-up of patients with inflammatory bowel diseases (IBD) requires the clearest possible picture of the immunological mechanisms underlying these complex pathologies. We identified recently a potential new player in this destructive game, a non-conventional T cell subset called Mucosal-Associated Invariant T (MAIT) cells. These cells were initially identified on the basis of their use of a semi-invariant TCR, made of the invariant Vα7.2–Jα33 TCRα chain (now TCRAV1S2–AJ33) paired to a limited number of different TCRβ chains (1). Human MAIT cells are mostly CD8+ T cells with an effector/memory phenotype and expression of various chemokine receptors involved in extra-lymphoid migration. They also express most markers associated with IL-17 producing T cells, such as RORγt, high CD161, IL-23R, and CD26. They make up to 10% of peripheral blood and intestinal lamina propria T cells, and are even more abundant in the liver (2). The most striking feature of MAIT cells is their recognition of highly conserved microbial-derived metabolites associated to a monomorphic MHC class-I like molecule, MR1 (MHC-related 1) (3). These ligands structurally belong to the pterin family and are derived from the riboflavin synthesis pathway. Recent experiments showed that virtually all MAIT cells are stained by fluorescent MR1 tetramers loaded with these specific metabolites. This pathway is absent in vertebrates, but many bacterial and fungal species produce riboflavin and therefore, MAIT cells-specific ligands. In this respect, these metabolites behave like microbial innate signals, and may alert MAIT cells that an invasive infection is on-going. Upon activation, MAIT cells release TNFα, IFNγ and become cytotoxic; they also produce IL-17 in specific conditions. In fact, they represent the great majority of naturally occurring IL-17-producing CD8+ T cells in the human peripheral blood. These cells are very likely to perform important anti-microbial functions, as suggested in humans and mice models (4). However, numerous reports suggest that MAIT cells are recruited from the blood to inflamed tissues in chronic inflammatory diseases such as multiple sclerosis, psoriasis, and systemic lupus, among others. We recently showed that Crohn’s disease (CD) patients display a decreased number of blood MAIT, balanced by their accumulation in the inflamed portions of the gut (5). As already stated, MAIT cells are equipped with chemokine receptors allowing migration toward tissues, in particular in conditions of inflammation. Therefore, it might be suggested that they are non-specifically attracted to sites of inflammation and are only bystanders in this process. However, we wish to discuss in this opinion article the arguments in favor of a relevant role for this T cell subset, at least in the context of CD.

We showed in our study that blood MAIT cells from CD patients showed an altered phenotype, increased *in vivo* proliferation, and, interestingly, a shift in cytokine production with decreased IFNγ and increased IL-17 production (5). While this description does not allow any formal conclusions about the direct involvement of MAIT cells in the pathophysiology of the disease, it has several important implications. Indeed, although blood MAIT cells may be non-specifically attracted to the inflamed gut by locally produced chemokines, it must be reminded that a significant number of them are found in the gut lamina propria in the healthy intestine. Therefore, it is more than likely that these intestinal cells are also activated in CD, and produce cytokines, which are highly relevant to the pathology, i.e., IFNγ, TNFα, and IL-17. Hence, it is difficult to suggest that these local cells (as well as newcomers from blood), strongly activated and producing inflammatory cytokines, have no consequences on the local inflammation. The question that needs to be addressed is the mechanisms by which MAIT cells may be activated; we propose two non-mutually exclusive hypotheses:

1. The microbial ligands recognized by the MAIT cells TCR (named RL antigens after their ribityl-lumazine composition) derive from a conserved pathway of riboflavin synthesis. This pathway is conserved among many bacterial species, and may explain MAIT cells activation and recruitment in response to infections by *Salmonella typhimurium*, *Mycobacterium tuberculosis*, *Shigella flexneri*, *Vibrio cholerae*, and others. However, these ligands are also synthetized by non-pathogenic bacteria, including several species found in the normal gut microbiota. In mice, peripheral maturation of MAIT cells is dependent upon the colonization of the intestine by the commensal flora. Therefore, there are strong interactions between MAIT cells and ligands derived from the commensal flora. Nevertheless, mechanisms must exist to ensure that intestinal MAIT cells do not aggressively respond to gut bacteria in the steady-state. In IBD, the disruption in local immune homeostasis results in dysbiosis, increased permeability of the mucosa, and subsequent increased bacterial translocation. Therefore, it is likely that these events induce also an increased availability of antigens derived from the commensal flora and therefore, MAIT cells activation.
2. Human MAIT cells constitutively express receptors for IL-12, IL-23, and IL-18. It is now demonstrated that they can be activated in the absence of their cognate antigens by a combination of IL-12 + IL-18 (6). MAIT cells activation by cytokines result in exclusive IFNγ secretion, at least in the blood and liver (7). Furthermore, both cytokines play an important function in MAIT cells activation by bacteria, independently of their capacity to produce riboflavin, since blocking antibodies to IL-12 and IL-18 strongly inhibit activation. This pathway depends on activation of the inflammasome in monocytes, resulting in cytokine secretion and MAIT cells activation in co-culture experiments. IL-12 and IL-18 are both over-produced in the intestinal mucosa of CD patients. IL-23R, NLRP3, IL-18R1, and IL12B2 gene polymorphisms are significantly associated with CD, further suggesting a prominent role for these cytokines in the pathophysiology of the disease. Therefore, it is likely that this cytokine environment participates locally in MAIT cells activation, and may influence their survival and/or proliferation. Altogether, the cytokines produced in large quantities by intestinal antigen-presenting cells create an environment that favors MAIT cells activation, both in the presence and absence of their cognate antigens.

## Dr. Jekyll or Mister Hyde?

The next important question is obviously the role that activated MAIT cells may play during CD. In most reports describing MAIT cells implications in autoimmune diseases, the authors suggest that these cells are pro-inflammatory and deleterious. This is inferred from the fact that *in vitro* mitogen-activated MAIT cells produce IFNγ, TNFα, and IL-17. Given the reported inflammatory role for IL-17 in several chronic inflammatory diseases, this cytokine secretion pattern is interpreted strictly as pro-inflammatory. However, it is important to pinpoint several facts. First, in most reports, MAIT cells activation *in vitro* by riboflavin-producing bacteria induce mostly IFNγ and TNFα secretion, with little or no IL-17. Accordingly, cytokines-induced MAIT cells activation results solely in IFNγ production. Second, very little is known about the regulation of cytokine secretion by MAIT cells *in vivo*. Of note, there is no published description of the functional response of mucosal MAIT cells to bacteria, their cognate antigens and/or cytokines. It is likely that the mucosal specific environment (cytokines, antigen-presenting cells, and other features) skews MAIT cells functions, as already described for conventional T cells. This question is of great importance in the context of CD, where the major inflammatory cytokine is suggested to be IFNγ, although IL-23 and IL-17 are produced in great quantities in the inflamed mucosa (8). Indeed, several studies reported a protective role for IL-17 in the intestinal mucosa (9, 10), and clinical trials with the anti-IL-17 antibody secukinumab demonstrated an absence of efficiency in CD (11), suggesting IL-17 is not the major cytokine in the inflammatory process (12). In our own study, we reported that blood MAIT cells from CD patients show a shift in cytokine secretion with lower IFNγ and higher IL-17. Therefore, it is possible that in CD, MAIT cells recruited from the circulation to the mucosa display enhanced protective IL-17 secretion in response to the local inflammation, as an endeavor to heal the aggressed mucosa. In other words, there is insufficient data available to demonstrate at this point that MAIT cells are necessarily pro-inflammatory, especially in the context of IBD.

## Concluding Remarks

There is no demonstration to date that MAIT cells are directly involved in autoimmune/inflammatory diseases. However, there is sufficient data to foster more studies on this topic, especially in CD. The main problem is that there is no relevant animal model available, as mice display a defect in MAIT cells development. Therefore, it is important to pursue the analysis of human MAIT cells *in vitro* and *ex vivo*, in healthy donors and CD patients. There is a need to study these cells in the mucosa, where they probably behave differently than at the systemic level. It will be also useful to analyze the role that some important gene polymorphisms involved in CD may have on MAIT cells activation and functional response. Finally, longitudinal analysis of MAIT cells phenotype and functions in CD patients undergoing various targeted therapies will also shed light on their role in the disease, and, possibly, their potential use as biomarkers.

## Conflict of Interest Statement

The author declares that the research was conducted in the absence of any commercial or financial relationships that could be construed as a potential conflict of interest.
